# New Tricks with an Old Sponge: Feature-Based Molecular Networking Led to Fast Identification of New Stylissamide L from *Stylissa caribica*

**DOI:** 10.3390/md18090443

**Published:** 2020-08-27

**Authors:** Silvia Scarpato, Roberta Teta, Gerardo Della Sala, Joseph R. Pawlik, Valeria Costantino, Alfonso Mangoni

**Affiliations:** 1 Dipartimento di Farmacia, Università degli Studi di Napoli Federico II, via Domenico Montesano 49, 80131 Napoli, Italy; silvia.scarpato@unina.it (S.S.); roberta.teta@unina.it (R.T.); valeria.costantino@unina.it (V.C.); 2 Laboratory of Pre-Clinical and Translational Research, IRCCS-CROB, Referral Cancer Center of Basilicata, 85028 Rionero in Vulture, Italy; gerardo.dellasala@crob.it; 3 Department of Biology and Marine Biology, University of North Carolina Wilmington, Center for Marine Science, 5600 Marvin K Moss Lane, Wilmington, NC 28409, USA; pawlikj@uncw.edu

**Keywords:** cyclic peptides, dereplication, feature-based molecular networking, marine sponges, metabolomics, molecular networking, proline-rich peptides

## Abstract

Feature-based molecular networking was used to re-examine the secondary metabolites in extracts of a very well studied marine sponge, *Stylissa caribica*, known to contain a large array of cyclic peptides and brominated alkaloids. The analysis revealed the presence of 13 cyclic peptides in the sponge that had never been detected in previous work and appeared to be new compounds. The most abundant one was isolated and shown to be a new proline-rich cyclic heptapetide that was called stylissamide L (**1**). Structure of compound **1**, including the cis/trans geometry of the three proline residues, was determined by extensive NMR studies; the l configuration of the seven amino acid residues was determined using Marfey’s method. Stylissamide L was tested for activity as a cell growth inhibitor and cell migration inhibitor on two cancer cell lines but, unlike other members of the stylissamide family, it showed no significant activity. This approach showed that even a thoroughly studied species such as *S. caribica* may contain new chemistry that can be revealed if studied with the right tools.

## 1. Introduction

Marine organisms are currently the most prolific source of chemically diverse natural products [1,2], with a wide range of pharmacological activities, including anticancer [3] and antibiotic properties [4]. After the first pioneering years, when only abundant or easy-to-collect species were studied extensively, the interest of many marine chemists has moved towards organisms that are endemic to limited areas, live in exotic environments such as polar seas or hydrothermal vents, or are hard to collect in sufficient amounts, and this exploration is far from being concluded. Still, even a well-known and extensively studied species may contain new chemistry that has yet to be discovered, provided that suitable tools are used.

The bottleneck in natural product discovery is no longer structure elucidation. Modern methods for structure elucidation are fast and sensitive, and in most cases full elucidation of structure and stereochemistry can be achieved with a few micrograms of sample [5]. The current challenge in natural product research is the rapid identification of new natural products in complex extracts, that may contain hundreds or thousands of different compounds, including primary metabolites, known natural products, or contaminants from the isolation. This process is usually called dereplication, giving the word a broader sense than its original meaning (early identification of known natural products) [6].

Dereplication can be best approached using liquid chromatography coupled with tandem mass spectrometry (LC-MS^2^), and particularly high resolution LC-MS^2^ (LC-HRMS^2^). These techniques provide huge amounts of data, which is adequate to the complexity of the systems under study, but prevents any efficient data analysis based on visual inspection. Therefore, many bioinformatic methods for the analysis of the results of LC-MS^2^ experiments have been developed. Among them, an increasingly important role is being played by molecular networking, a modern computational approach for the automated identification of structural similarity between compounds, inferred by the relatedness of their MS^2^ spectra [7]. Molecular networking has been shown to give remarkably better results if LC-MS^2^ raw data are preprocessed before network generation using tools such as MZmine [8] or OpenMS [9], which can detect MS features such as isotope patterns and LC features such as retention times and peak areas. This protocol is referred to as Feature-Based Molecular Networking (FBMN) [10], and has been recently implemented and documented in the main online platform for molecular networking, GNPS [11], but can also be implemented locally using the stand-alone molecular networking program MetGem [12] together with MZmine and the visualization program Cytoscape [13].

The value of molecular networking in natural product research has been recently demonstrated by the isolation of two new compounds belonging to the smenamide family, smenamide F and G from the extract of the sponge *Smenospongia aurea* [14], by the discovery of the new cytotoxic saponin holothurin A5 from the sea cucumber *Holothuria atra* [15], and by the detection of five new congeners of thermoactinoamide A (thermoactinoamide G–K) from the extract of the bacterium *Thermoactinomyces vulgaris* [16]. In the present study, we used molecular networking to examine the extract of *Stylissa caribica.*

The marine sponge *S. caribica* has been thoroughly studied and shown to be very rich in secondary metabolites. Twenty different brominated pyrrole-imidazole alkaloids have been detected in *S. caribica* so far, and nine of them have been reported for the first time from this sponge, including N-methyldibromoisophakellin, oxocyclostylidol, 4-bromopyrrole-2-carboxy-*N*(ε)-lysine, and 4-bromopyrrole-2-carboxyarginine. In addition, 13 cyclic heptapeptides have been found in *S. caribica* [17,18,19,20,21], and ten of them were new compounds at the time of the report (Table S1). Among them, it is worth mentioning stylissamide G and H, which exhibited cytotoxic activity towards HCT-116, human colon tumor cell line [21].

**Figure 1:**
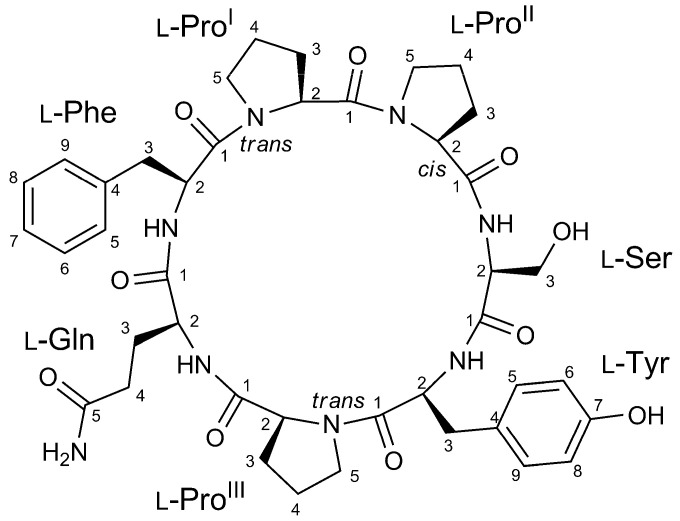
Structure of stylissamide L (**1**).

In spite of the thorough analyses of previous studies, the molecular networking approach led to the straightforward identification of a new natural product from *S. caribica*, namely the cyclic heptapeptide stylissamide L (**1**) (Figure 1). The identification and isolation of stylissamide L, its structural elucidation by MS, NMR, and Marfey’s analysis, and the examination of its biological activity are here described.

## 2. Results and Discussion

### 2.1. Collection, Extraction, LC-MS^2^ Analysis, and Costruction of the Molecular Network

A sample of the marine sponge *Stylissa caribica*, collected along the coast of Compass Cay Island, in the Exuma Islands (Bahamas Islands), was extracted with MeOH and CHCl_3_ mixtures. The MeOH extract was partitioned between H_2_O and *n*-BuOH, and the *n*-BuOH layer was combined with the other organic extracts. The total organic extract was chromatographed using a reversed-phase column chromatography on RP-18 silica gel.

One way to improve the quality of untargeted metabolic profiling is to use a wider separation space, which helps to keep the number of co-eluting metabolites low. Therefore, RP-18 fractions (rather than the crude organic extract) were analyzed by liquid chromatography coupled with high-resolution tandem mass spectrometry (LC-HRMS^2^) performed using an LTQ Orbitrap instrument with an electrospray (ESI) source and a pentafluorophenyl (PFP) high-performance liquid chromatography (HPLC) column, because of its partially orthogonal retention ability compared to the RP-18 stationary phase. After each full MS scan, the five most intense ions in the spectrum were fragmented in subsequent MS^2^ scans. From these data, a molecular network was generated by combined use of MZmine2 and MetGem.

The preprocessing of LC-MS^2^ data with MZmine was the key for obtaining a clear and informative network and will be discussed in some detail here. In our view, the final goal of FBMN is to achieve the equality one node, one compound. In classical molecular networking, this goal is prevented by a number of obstacles. On one hand, the same compounds can give rise to more nodes, because of the presence of the isotope peaks and the frequent formation of different adduct ions (e.g., [M+H]^+^ and [M+Na]^+^), and because two noisy MS^2^ spectra can be mistaken as coming from different compounds when MS^2^ spectra are clustered. On the other hand, isomeric compounds can collapse into the same node if they show similar MS^2^ spectra and chromatographic information is not taken into account. To circumvent these problems, the following scheme for the preprocessing of LC-MS^2^ data was used.

After standard initial data processing (mass detection, chromatogram build, and chromatogram deconvolution), data from the LC-MS^2^ runs of individual fractions were joined in a single feature list using the Join aligner module. The *Adduct search* module was then used not only to identify peaks of [M+Na]^+^, [M+NH_3_]^+^, and [M+K]^+^ adduct ions, but also to identify ^13^C (mass difference 1.0033) and ^81^Br (mass difference 1.9979) isotope peaks. They were subsequently all removed using the *Row filter* module. As a result, most compounds in the extract gave only a single entry in the feature list. Finally, the *Export to GNPS* module was used to export the MS^2^ spectra into an .mgf file and quantitative data into a .csv file, which were used for the construction of the molecular network. Detailed information of data processing can be found in Stylissa_MZmine.xml in the Supplementary Materials section.

Construction of a molecular network requires the selection of a few parameters that can dramatically affect the resulting network, and whose optimal values are strongly dependent on the nature of the sample, on the technology of the MS instrument, and on the settings used for the LC-MS^2^ runs. The three most important networking parameters are the mass tolerance for peak matching, the minimum number of matched peaks for a cosine score to be calculated, and the minimum cosine score for two nodes to be connected. Optimization of these parameters was pursued using the program MetGem, which for small datasets is far faster than the GNPS website (a few seconds vs. at least a few minutes). We found that setting the mass tolerance to 0.01 Da for both the parent and the fragment ions, the minimum number of matched peaks to eight, and the minimum cosine score to 0.55 produced the largest and most informative set of clusters, while still keeping the number of false positives low.

The .mgf and .csv files were then submitted to the GNPS website to produce the final, public version of the network. The Dereplicator tool in GNPS was then used to identify some of the nodes in the network. Unexpectedly, the network obtained using the new Feature-Based Molecular Networking workflow, combined with the optimized parameters discussed above was remarkably different compared to the network produced by MetGem, with smaller clusters and many missing nodes (including the node of stylissamide L) (Figure S1 and Table S3). Contrarily, the network produced using the older Metabolomics workflow and the same parameters was identical to the MetGem network. We were not able to determine the reason for this unexpected outcome and proceeded with the Metabolomics workflow. The feature-based network was constructed and visualized using the Cytoscape software importing the relevant features directly from the quantitation file exported from MZmine.

**Figure 2:**
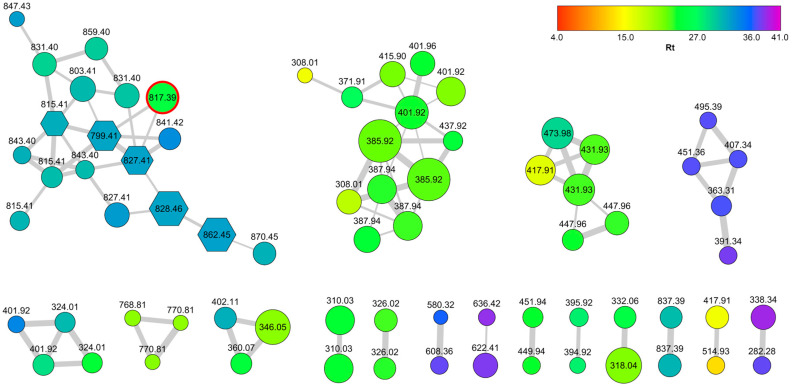
The feature-based molecular network of *S. caribica*. Nodes are color-coded according to retention times, and their size is related to the amounts of the metabolite. Annotated nodes are shown as hexagons and can be identified using Table S1. The node of Stylissamide L (1) is marked with red borders.

The resulting network is shown in Figure 2. In the network, the color of each node is mapped to the relevant retention time to give a visual indication of the polarity of the metabolite, and the size of the node is related to the amounts of the metabolite. In addition, nodes annotated by Dereplicator with a putatively identified metabolite are represented as hexagons.

Most clusters in the network were related to brominated compounds, which are abundant and diverse in *S. caribica*, but the largest cluster in the network was the cluster of cyclic peptides. Five of the nodes in this cluster could be putatively annotated as known peptides, two of which were not previously reported from *S. caribica* (Table S1), but the remaining 13 nodes could not be associated with any known natural peptide, indicating the presence of new compounds. Interestingly, the most abundant unknown peptide (*m*/*z* 817.39) showed a much shorter retention time compared with the other peptides in the cluster; it was not present in the RP-18 fraction (fraction F4) where most of the other peptides were eluted, but in the earlier fraction F3. This peptide was isolated as a pure compound (7.2 mg) in a single step of reversed-phase HPLC chromatography and named stylissamide L (**1**).

### 2.2. Structure Elucidation of Stylissamide L (**1**)

The high resolution ESI mass spectrum of stylissamide L (**1**) showed [M+H]^+^ and [M+Na]^+^ ion peaks at *m*/*z* 817.3876 and *m*/*z* 839.3694, respectively, which defined its molecular formula as C_41_H_53_O_10_N_8_ with 20 unsaturations. The fragmentation pattern observed in the MS^2^ spectrum of compound **1** confirmed a cyclic peptide structure, with fragments originating from the loss of H_2_O and CO and of one phenylalanine, one glutamine, one tyrosine, and one proline residues. The molecular formula was satisfied with the presence of one serine and two further proline residues in addition to the four residues above, thus defining the amino acid composition of compound **1**, which was later confirmed by NMR analysis. Considering that these seven amino acids accounted for 19 degrees of unsaturation, the 20 unsaturations determined by the molecular formula confirmed the cyclic structure of compound **1**.

A full set of homonuclear and heteronuclear two-dimensional NMR spectra (COSY, TOCSY, NOESY, HSQC, and HMBC) were recorded (Figures S3–S11). The proton spectrum showed four amide NH signals and seven α-proton signals, as expected for a cyclic heptapeptide with three proline residues. The aliphatic protons of each residue were identified from their cross peaks with the corresponding α-proton or amide NH signals in the TOCSY spectrum, and their assignment was achieved using the COSY and HSQC spectra (Table 1 and Figure S8).

**Figure 3:**
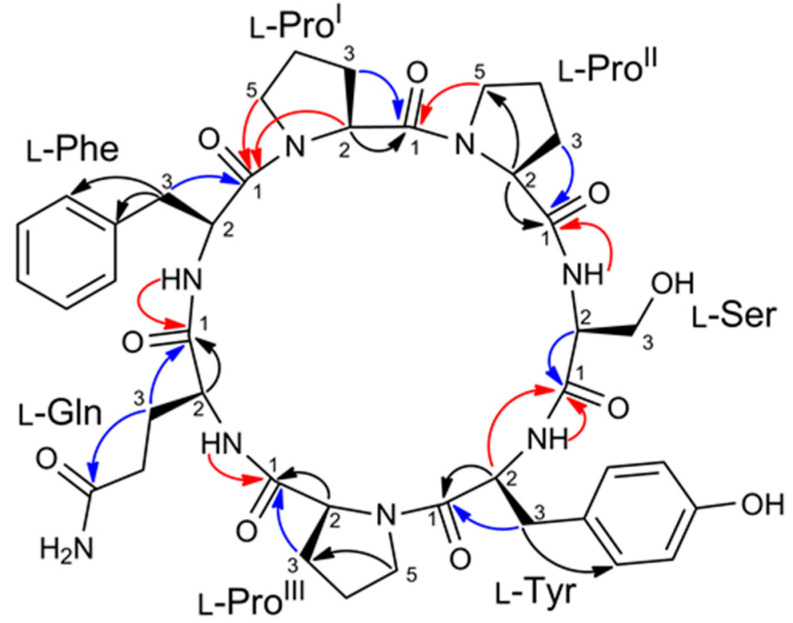
Diagnostic HMBC correlation used to elucidate amino acid sequence in stylissamide L (**1**). Correlations used to assign carbonyl ^13^C signals are noted with blue arrows, intra-residual correlations are noted with red arrows.

The amino acid sequence in the peptide was determined from HMBC data. In addition to the standard HMBC experiment, a band selective HMBC experiment was used to improve resolution in the ^13^C dimension and allow for discrimination of CO signals with very close ^13^C chemical shifts such as Pro^II^-C1 and Pro^III^-C1 (Figure S12). The most significant HMBC correlations used to elucidate the amino acid sequence are shown in Figure 3. The carbonyl ^13^C signals of each amino acid were assigned (except for Ser) based on their HMBC correlations with one or both protons at the respective β methylene (i.e., at position 3) (blue arrows in Figure 3). Inter-residue linkages were established by the HMBC correlations of the four amide protons (Ser-NH with Pro^II^-C1, Tyr-NH with Ser-C1, Gln-NH with Pro^III^-C1, Phe-NH with Gln-C1) and of proline ε protons (Pro^I^-5b with Phe-C1 and Pro^II^-5b with Pro^I^-C1) (red arrows in Figure 3), this defining the sequence as cyclo (Pro-Pro-Ser-Tyr-Pro-Gln-Phe).

The absolute configuration of the seven amino acid residues was defined by an advanced Marfey’s methodology, using the Orbitrap high-resolution MS instrument as detector to improve sensitivity and specificity and perform the analysis using only a few µg of sample [5,22]. Compound **1** (32 μg) was subjected to total hydrolysis by treating it with 6 N HCl/AcOH (1:1) at 120 °C for 18 h and then derivatized with the d-enantiomer of Marfey’s reagent (1-fluoro-2-4-dinitrophenyl-5-d-alanine amide, or d-FDAA), adding 100 μL of 1% d-FDAA. In the total hydrolysis conditions used, the glutamine residue is transformed into glutamic acid. The resulting d-FDAA derivatives of Pro, Ser, Tyr, Glu and Phe were analyzed by high-resolution LC-MS, and their retention times were compared with authentic standards prepared by reaction of l- and d-FDAA with l-Pro, d-Ser, l-Tyr, l-Glu, l-Phe. LC-MS analysis revealed the l configuration for all amino acids, based on the retention times of Marfey’s derivatives; the exclusive presence of l amino acids was in accordance with the other heptacyclopeptides of the stylissamide class.

The NOESY spectrum of stylissamide L (**1**) showed many cross peaks between topologically far protons (e.g., Tyr-NH with Phe-NH or Tyr-NH with Pro^I^-H2; see also Table S2) suggesting a highly structured conformation as in other stylissamides [21]. The electronic circular dichroism (ECD) spectrum (Figure S13) showed a quite complex band structure, with a positive Cotton effect at 236 nm and negative Cotton effects at 219 and 202 nm. It has been shown that configurational isomerism about proline peptide bonds is possible in strained cyclic peptides like, for example, for stylissamide H and euryjanicin A [21]. Therefore, the cis or trans geometry of the bond of proline residues with the preceding amino acid should be considered a configuration rather than a conformation in such compounds, and needed to be clarified to complete structural elucidation of stylissamide L. Pro^II^ was determined to be cis because of the NOESY cross peak between Pro^II^-H2 and Pro^I^-H2, and because the difference between the ^13^C NMR chemical shift of Pro^II^-C3 and Pro^II^-C4 was greater than 8.0 ppm, with Pro^II^-C4 below 23.3 ppm, in accordance with an empirical rule discussed in ref. [19]. Likewise, Pro^I^ and Pro^III^ were deduced to be trans because the respective differences (3.8 and 3.7 ppm) between C-3 and C-4 chemical shifts were well below the 8.0 ppm threshold. Additionally, no NOESY cross peaks conflicting with this assignment were detected.

From the structural point of view, stylissamide L is analogous in many ways to the other members of the family of stylissamide, which are all heptapeptides rich in proline (from two to four proline residues); however, it is the first example of a stylissamide containing a serine residue. The reason why stylissamide L is poorly retained by RP-18 stationary phase has no easy explanation. Stylissamide L lacks aliphatic amino acids other than proline, but this feature is common to other analogues like stylissamide F, which showed remarkably longer retention times; on the other hand, compounds with apparently similar polarity, like stylissamide A, are retained even less than stylissamide L by the RP-18 stationary phase (Table S1). It is possible that RP-18 retention times may be strongly dependent on the conformation of the peptide, which may prevent non-polar regions of the molecule from interacting with the hydrophobic chromatographic stationary phase.

### 2.3. Cell Proliferation and Migration Assays

The peculiar conformational features of stylissamide L and the cytotoxic activity reported for some stylissamides prompted evaluation of the growth inhibitory effects of stylissamide L (**1**). Assays were conducted using MCF-7 breast cancer and BxPC-3 pancreatic cancer cells, through impedance-based dynamic monitoring of cell proliferation after drug exposure, following a previously described procedure [23]. After 72 h incubation with different concentrations (6.25, 12.5, 25, and 50 µM) of **1**, MCF-7 and BxPC-3 cell growth remained substantially unaffected even at the highest dose tested (Figure S14).

Based upon structure similarity with the known cell-migration inhibitor stylissamide X [24], stylissamide L (**1**) was then evaluated for its ability to affect cell motility. Cell migration consists of chemoattractant-induced movement of cells from one location to another and is a crucial step in tumour cell dissemination and formation of metastases, making it an attractive target in cancer therapy. Migration of MCF-7 breast cancer cells and 3AB-OS osteosarcoma stem cells was monitored for 20 h after exposure to 10 and 50 µM of compound **1**. Migratory activity of MCF-7 and 3AB-OS cells was unaffected or even slightly increased at 50 µM of **1** (Figure S15). 

In spite of the disappointing results of the assays described above, the structural diversity of the cyclic heptapeptides found in *Stylissa* sponges and the biological activity shown by some of them makes this group of metabolites worthy of further examination. A more complete study about the biological activity of all cyclic peptides isolated from *S. caribica*, also aimed to determine the structure–activity relationship, is in progress and the results will be reported in the due course.

## 3. Materials and Methods

### 3.1. General Experimental Procedures

A Jasco P-2000 polarimeter (Jasco Europe s.r.l., Cremella, Italy) at the sodium D line was used to measure optical rotations. ^1^H NMR and 2D NMR experiments were carried out at 700 MHz on a Bruker Avance Neo spectrometer (Bruker BioSpin Corporation, Billerica, MA, USA) using dimethylsulfoxide-*d*_6_ (DMSO-*d*_6_) as solvent; all chemical shifts were referenced to the residual solvent signal (δ_H_ 2.50, δ_C_ 39.5). The HSQC spectra were optimized for ^1^*J*_CH_ = 142 Hz and the HMBC experiments for ^2,3^*J*_CH_ = 8.3 Hz. Through-space ^1^H connectivities were evidenced using a NOESY experiment with a mixing time of 300 ms. High-resolution MS and LC-MS experiments were recorded on a Thermo LTQ Orbitrap XL mass spectrometer (Thermo Fisher Scientific Inc., Waltham, MA, USA) combined to a Thermo U3000 HPLC system. High-performance liquid chromatography (HPLC) separations were achieved on an Agilent 1260 Infinity Quaternary LC apparatus (Agilent Technology, Cernusco sul Naviglio, Italy), equipped with a diode-array detector (DAD).

### 3.2. Collection, Extraction and Isolation

The sample of *Stylissa caribica,* investigated in this study, was collected at 28 m depth by Scuba along the coast of Compass Cay Island, in the Exuma Island of the Bahamas (GPS 24° 16.372′ N, 76° 30.141′ W) during a ship-based research expedition in 2010. After collection, the sponge was immediately frozen and kept at −20 °C until extraction, which was performed using our standardized procedure [25]. Briefly, the frozen sponge (154 g wet weight) was chopped into small pieces and extracted at room temperature with MeOH (4 × 1.5 L), mixtures of MeOH and CHCl_3_ in different ratios (2:1, 1:1, 1:2) and then with CHCl_3_ (2 × 1.5 L). The orange colored MeOH extract was partitioned between H_2_O and *n*-BuOH. The resulting *n*-BuOH layer was merged with the CHCl_3_ extracts and dried under vacuum.

The total organic extract (8.08 g) was chromatographed using a reversed-phase column chromatography on RP-18 silica gel. Fractions F3 (eluted with 60% MeOH, 1060 mg), F4 (80% MeOH, 830 mg), F5 (90% MeOH, 410 mg), and F6 (MeOH/CHCl_3_ (9:1), 460 mg) were used for LC-MS^2^ analysis (see below). Fraction F3, containing stylissamide L (**1**), was subjected to reversed-phase HPLC separation on a Luna (Phenomenex) C18 column (250 × 10 mm, 10 μm) (Eluent A: 0.1% HCOOH in H_2_O; eluent B: MeOH; gradient program: 25% B 5 min, 25% → 50% B over 27 min, 50% → 100% B over 3 min, 100% B 7 min; flow rate 5 mL min^–1^, wavelength 230 nm) to afford a fraction (*tR* = 24 min) containing 7.2 mg of pure compound **1**.

*Stylissamide L* (**1**): light yellow powder; ${\left[\alpha \right]}_{D}^{20}$–40 (c 0.23, acetonitrile); UV (ACN): λ_max_ (ε) 277 (1250), 232 (5900, shoulder), 195 (50500); ECD (ACN): λ_max_ (Δε) 236 (+4.9), 219 (−15.3), 202 (−18.6); high resolution ESI-MS (positive ion mode, MeOH) *m*/*z* 817.3876 ([M + H]^+^, C_41_H_53_O_10_N_8_^+^, calcd. 817.3879), 839.3694 ([M + Na]^+^, C_41_H_52_O_10_N_8_Na^+^, calcd. 839.3699). ^1^H and ^13^C NMR (DMSO-d_6_): Table 1.

### 3.3. LC-HRMS and LC-HRMS^2^

All LC-HRMS and LC-HRMS^2^ analyses were performed on a Thermo LTQ Orbitrap XL high-resolution ESI mass spectrometer coupled to a Thermo U3000 HPLC system. Experiments were performed with a Kinetex 5 µm, 100 mm × 2.1 mm PFP column (Phenomenex, Torrance, CA, USA), kept at 25 °C, using an elution gradient of H_2_O and MeOH running and a flow rate of 200 μL/min. The gradient program was as follows: 10% MeOH for 1 min, 10−100% MeOH over 30 min, and 100% MeOH for 10 min. Mass spectra were acquired in positive ion detection mode, with resolution set to 60,000 in the range of m/z 150–2000. MS parameters were set as follows: a spray voltage of 4.80 kV, a capillary temperature of 285 °C, a sheath gas rate of 32 units N_2_ (ca. 320 mL/min), and an auxiliary gas rate of 15 units N_2_ (ca. 150 mL/min). Data were recorded with data-dependent acquisition (DDA) mode, in which the four most intense ions in the full-scan mass spectrum were subjected to high resolution tandem mass spectrometry (HRMS^2^) analysis. HRMS^2^ scans were achieved for selected ions with collision induced dissociation (CID) fragmentation, an isolation width of 3.00 Da, a normalized collision energy of 35 units, an activation Q of 0.250 units, and an activation time of 30 ms. Mass data were analyzed using the Thermo Xcalibur software version 2.2 (Thermo Fisher Scientific Inc., Waltham, MA, USA).

### 3.4. LC-HRMS^2^ Data Processing and Molecular Networking

Raw LC-HRMS^2^ data were processed in batch mode with the software MZmine version 2.51 [8]. The batch queue used for processing was saved in the file sytlissa_MZmine.xml that is reported in the Supplementary Materials. Mass spectrometry data were deposited on MassIVE (accession number: MSV000085867). Molecular networking was performed using MetGem version 1.2.2 [12] and/or the GNPS website [11] with the same parameters: *m*/*z* tolerance 0.01 Da, cosine score > 0.55, matched peaks > 8, maximum number of neighbor nodes = 10, maximum number of nodes in a single network = 100. The generated network was visually displayed with Cytoscape version 3.7.1 [13], and the relevant features were mapped to each node by importing the quantitation file generated from MZmine. Dereplication of known compounds was performed on GNPS, using the DEREPLICATOR V2 algorithm, setting a precursor ion mass tolerance and a fragment ion mass tolerance of 0.02 Da. Links to deposited LC-MS data and molecular networks are listed in Table S3.

### 3.5. Advanced Marfey’s Analysis

An amount of 32 μg of stylissamide L (compound **1**) was hydrolyzed with 500 μL 6 N HCl/AcOH (1:1) at 120 °C for 18 h. The residual HCl fumes were removed under a direct N_2_ flux. The hydrolysate of **1** was dissolved in TEA/acetone (2:3, 100 μL) and 1% 1-fluoro-2,4-dinitrophenyl-5-d-alaninamide (d-FDAA) in CH_3_CN/acetone (1:2) (100 μL) was added. The mixture was heated at 50 °C for 2 h and dried under N_2_ stream. It is important to note that in the hydrolysis conditions used the glutamine residue is transformed into glutamic acid. The resulting d-FDAA derivatives of all amino acids (Pro, Ser, Tyr, Gln, Phe) were dissolved in MeOH (100 μL) for subsequent analysis. Authentic standards of l-Pro, d-Ser, l-Tyr, l-Glu and l-Phe were treated with l-FDAA and d-FDAA using the same procedure described above. The retention times of Marfey’s derivatives of compound **1** were compared with those of the standard derivatives by LC-HRMS^2^ using a Kinetex C18 (Phenomenex) 150 × 2.1 mm, 5 μm column. The gradient conditions were set as follows: 35 min prerun with 5% ACN, 5% ACN 3 min, 5% → 50% ACN over 30 min, 50% ACN 1 min, 50% → 90% ACN 1 min, 90% ACN 6 min. Mass spectra were acquired in positive ion detection mode, and raw data were analyzed using the Xcalibur suite of programs.

### 3.6. Cell Proliferation and Migration Assays

Cell proliferation assays were performed using the xCELLigence System Real-Time Cell Analyzer (ACEA Biosciences, San Diego, CA, USA), as previously described [23].

Migration activity was also evaluated by the xCELLigence system, but, for this purpose, equipped with electronic cell invasion and migration plates (CIM-Plate 16). These plates are composed of upper and lower chambers, separated by a microporous membrane coated with gold microelectrodes, which display decreased electrical conductivity when cells adhere to their surface while moving towards the lower chamber. For the migration assay, 5.0 × 10^4^ cells/well were seeded in the upper chamber with stylissamide L (**1**) or 0.1% DMSO vehicle, in a serum-free growth medium. The lower chambers were filled with growth medium supplemented with the chemoattractant 10% Fetal Bovine Serum (Gibco-Thermo Scientific, Waltham, MA, USA) or without it (negative control). Cell migration was monitored every 15 min for 20 h, through real time measurement of electronic impedance variations recorded by the microelectrodes located on the lower side of the microporous membrane. Data were analyzed by the Real-Time Cell Analyzer (RTCA)-integrated software (Version 2.0.0.1301, ACEA Biosciences, San Diego, CA, USA).

## 4. Conclusions

Feature-based molecular networking allowed for the fast identification of stylissamide L (**1**), a new proline-rich cyclic heptapeptide, isolated from extracts of the sponge *S. caribica*. The method additionally revealed the presence of many potentially new minor cyclic peptides in *S. caribica*. Unexpectedly, the feature-based molecular networking workflow and the Metabolomics workflow on the GNPS online platform produced different results, starting from the same .mgf file and using the same networking parameters, with the Metabolomics workflow producing the same result as the standalone program MetGem. We are continuing to investigate this unexpected outcome. In spite of this problem, this work clearly showed that even a thoroughly studied sponge species such as *S. caribica* may contain plenty of new chemistry that can be revealed if studied with suitable tools such as feature-based molecular networking.

## Figures and Tables

**Table 1:** NMR data of stylissamide L (**1**) (^1^H 700 MHz, ^13^C 175 MHz, DMSO-*d*_6_).

AA	Pos.	δ_C_, Type		δ_H_, Mult (J in Hz)	AA	Pos.	δ_C,_ Type		δ_H_, Mult (J in Hz)
**Pro^I^**	1	170.3, C			**Pro^III^**	1	171.9, C		
	2	59.1, CH		4.34, dd (5.1, 8.6)		2	63.1, CH		4.06, t (8.7)
	3	28.1, CH_2_	a	2.15, m		3	28.7, CH_2_	a	2.22 m
			b	1.75, m				b	1.81, m
	4	24.3, CH_2_		1.87, m		4	25.0, CH_2_	a	2.11, m
	5	46.7, CH_2_	a	3.45, m				b	1.98, m
			b	3.36, m		5	46.9, CH_2_	a	3.93, ddd (6.8, 9.8, 9.8)
**Pro^II^**	1	171.8, C						b	3.82, m
	2	60.1, CH		4.28, dd (1.5, 8.8)	**Gln**	NH			8.17, d (7,0)
	3	31.8, CH_2_	a	2.16, m		1	170.7, C		
			b	2.00, m		2	52.8, CH		4.05, ddd (4.3, 7.0, 10.0)
	4	21.7, CH_2_	a	1.77, m		3	25.9, CH_2_	a	1.85, m
			b	1.57, m				b	1.73, m
	5	46.8, CH_2_	a	3.60, ddd (1.5, 8.4, 10.8)		4	31.5, CH_2_	a	2.13, ddd (7.2, 15.7, 7.2)
			b	3.33, ddd (10.8, 10.8, 7.1)				b	2.04, ddd (7.2, 15.7, 7.2)
**Ser**	NH			7.65, d (5.9)		5	174.5, C		
	1	167.7, C				5-NH_2_			6.92, s
	2	60.0, CH		3.85, ddd (3.6, 5.9, 10.2)	**Phe**	NH			7.11, d (7.2)
	3	60.9, CH_2_	a	3.46, dd (10.2, 11.9)		1	167.5, C		
			b	3.14, dd (11.9, 3.6)		2	51.5, CH		4.69, ddd (5.8,7.2, 8.0)
**Tyr**	NH			7.34, d (9.1)		3	36.9, CH_2_	a	3.18, dd (8.0, 14.2)
	1	171.5 C						b	2.71, dd (5.8, 14.2)
	2	51.5 CH		4.88 ddd (3.2, 9.1,10.9)		4	138.0, C		
	3	37.0 CH_2_	a	3.35, dd (3.2,13.5)		5/9	128.9, CH		7.16, d (7.5)
			b	2.42, dd (10.9, 13.5)		6/8	126.0, CH		7.18, t (7.3)
	4	126.6 C				7	128.0, CH		7.22, t (7.5)
	5/9	130.5 CH		7.08, d (8.5)					
	6/8	114.9 CH		6.66, d (8.5)					
	7	156.0 C							
	7-OH			7.42, s					

## Supplementary Materials

The following are available online at https://www.mdpi.com/1660-3397/18/9/443/s1. Table S1: cyclic heptapeptides found in *S. caribica*; Table S2, full NMR data of stylissamide L (1); Table S3: Links to deposited LC-MS^2^ data and molecular networks; Figure S1: molecular networks of *S. caribica* obtained using different workflows; Figures S2–S13: MS, MS^2^, 1D and 2D NMR, UV, and ECD spectra stylissamide L (1); Figures S14 and S15: evaluation of biological activity of stylissamide L (1); File Stylissa_MZmine.xml containing the processing parameters of LC-MS^2^ data from *S. caribica* fractions.
