# Supplementary material for: New Tricks with an Old Sponge: Feature-Based Molecular Networking Led to Fast Identification of New Stylissamide L from Stylissa caribica
Source: Mar Drugs. 2020 Aug 27;18(9):443. doi: 10.3390/md18090443 (PMC7551058; doi:10.3390/md18090443)
Supplement: Supplementary file 1 [file marinedrugs-18-00443-s001.zip › Stylissamide L - SM.pdf]

Supplementary Material

Table S1. Cyclic heptapeptides in the sponge *Stylissa caribica.*

Table S2. Full NMR data of stylissamide L (**1**) (^{1}H 700 MHz, ^{13}C 175 MHz, DMSO-d_6).

Table S3. Links to LC-MS data and molecular networks.

Figure S1. Molecular networks obtained using (a) the program MetGem (b) the Metabolomics workflow on GNPS, and (g) the Feature-Based Molecular Network workflow on GNPS.

Figure S2. Positive ion mode high-resolution ESI mass spectrum of stylissamide L (**1**).

Figure S3. Positive ion mode high-resolution ESI MS/MS spectrum of stylissamide L (**1**).

Figure S4. ^{1}H-NMR spectrum of stylissamide L (**1**) (700 MHz, DMSO-d_6).

Figure S5. ^{13}C-NMR spectrum of stylissamide L (**1**) (175 MHz, DMSO-d_6).

Figure S6. COSY spectrum of stylissamide L (**1**) (700 MHz, DMSO-d_6).

Figure S7. TOCSY spectrum of stylissamide L (**1**) (700 MHz, DMSO-d_6).

Figure S8. Spin systems of stylissamide L (**1**) from the sections of the TOCSY spectrum.

Figure S9. NOESY spectrum of stylissamide L (**1**) (700 MHz, DMSO-d_6).

Figure S10. HSQC spectrum of stylissamide L (**1**) (700 MHz, DMSO-d_6).

Figure S11. HMBC spectrum of stylissamide L (**1**) (700 MHz, DMSO-d_6).

Figure S12. Band-selective HMBC spectrum of stylissamide L (**1**) (700 MHz, DMSO-d_6).

Figure S13. UV and ECD spectra of stylissamide L (**1**) in ACN.

Figure S14. Real time monitoring of cancer cell proliferation after exposure to stylissamide L (**1**).

Figure S15. Real-time monitoring of 3AB-OS and MCF-7 cell migration after exposure to stylissamide L (**1**).

Table S1. Cyclic heptapeptides in the sponge *Stylissa caribica.*

Cyclic peptide	[M+H]+	Exact Mass	Retention time RP-18[a]	Retention time PFP[b]	Citation
Stylissamide A	$C_{44}H_{61}N_8O_9$	845.4556	1.72	26.34	*c,d,e*
Stylissamide B	$C_{44}H_{58}N_7O_8$	812.4341	ND	ND	*c,d,e*
Stylissamide C	$C_{48}H_{60}N_7O_8$	862.4498	20.29	32.66	*c,d,e*
Stylissamide D	$C_{45}H_{62}N_7O_8$	828.4654	19.55	32.48	*c,d,e*
Stylissamide E	$C_{39}H_{59}N_8O_9$	783.4400	13.29	28.76	*d,e*
Stylissamide F	$C_{43}H_{57}N_{10}O_9$	857.4304	13.94	32.21	*d,e*
Stylissamide G	$C_{45}H_{62}N_7O_7$	812.4705	ND	ND	*e*
Stylissamide H	$C_{44}H_{59}N_8O_8$	827.4450	ND	ND	*e*
Stylissamide L	$C_{41}H_{53}N_8O_{10}$	817.3876	6.21	24.95	this work
Hymenamide C	$C_{43}H_{55}N_8O_9$	827.4087	20.71	32.58	*f*
Hymenamide F	$C_{35}H_{61}N_{10}O_8S$	781.4389	1.67	26.40	*f*
Phakellistatin 3	$C_{42}H_{55}N_8O_9$	815.4087	18.50	31.89	*h*
Phakellistatin 13	$C_{42}H_{55}N_8O_8$	799.4137	20.09	32.47	*g*
Stylisin 1	$C_{45}H_{62}N_7O_8$	828.4654	ND	ND	*c,d,e,g*
Stylisin 2	$C_{44}H_{58}N_7O_8$	812.4341	ND	ND	*c,d,e,g*

a. Experiments were performed with a Kinetex 5 µm, 50 mm × 2.10 mm C18 column using a flow rate of 200 µL/min and the following elution gradient: 10% MeOH for 1 min, 10%–100% MeOH over 30 min, and 100% MeOH for 10 min.

b. Experiments were performed with a Kinetex 5 µm, 100 mm × 2.1 mm PFP column using a flow rate of 200 µL/min and the same elution gradient of H_2O and MeOH described above.

c. Schmidt, G.; Grube, A.; Köck, M. Stylissamides A-D - New proline-containing cyclic heptapeptides from the marine sponge Stylissa caribica. European *J. Org. Chem.* **2007**, 2, 4103–4110, doi:10.1002/ejoc.200700013.

d. Cychon C.; Köck,M. Stylissamides E and F, Cyclic Heptapeptides from the Caribbean Sponge Stylissa caribica. *J. Nat. Prod.* **2010,** *73,* 738–742, doi: 10.1021/np900664f.

e. Wang, X.; Morinaka, B. I.; Molinski, T. F. Structures and solution conformational dynamics of stylissamides G and H from the Bahamian Sponge Stylissa caribica. *J. Nat. Prod.* **2014**, *77*, 625–630, doi:10.1021/np400891s.

f. Grube, A.; Maier, T.; Köck, M. MS-guided Fractionation as a Fast Way to the Identification of New Natural Products – MALDI-TOF-MS Screening of the Marine Sponge Stylissa caribica. *Zeitschrift für Naturforsch. B* **2007**, *62*, 600–604, doi:10.1515/znb-2007-0420..

g. Mohammed, R.; Peng, J.; Kelly, M.; Hamann, M.T. Cyclic heptapeptides from the Jamaican sponge Stylissa caribica. *J. Nat. Prod.* **2006**, *69*, 1739–1744, doi:10.1021/np060006n.

h. This compound was not previously detected in specimens of *Stylissa caribica*. It was putatively identified by the Dereplicator tool in GNPS.

Table S2. Full NMR data of stylissamide L (1) (^{1}H 700 MHz, ^{13}C 175 MHz, DMSO-d_6).

AA	pos.	δ_C, type		δ_H, mult (*J* in Hz)	NOESY	HMBC
Pro^{I}	1	170.3, C		-		
	2	59.1, CH		4.34, dd (5.1, 8.6)	Ser-NH, Tyr-NH, Phe-NH	Phe-1
	3	28.1, CH_2	a	2.15, m	Pro^{II}-4b, Pro^{II}-3a	Pro^{I}-1
			b	1.75, m	Pro^{II}-2, Pro^{II}-4b	Pro^{I}-1
	4	24.3, CH_2		1.87, m		
	5	46.7, CH_2	a	3.45, m		
			b	3.36, m		Phe-1,Pro^{I}-2
Pro^{II}	1	171.8, C		-		
	2	60.1, CH		4.28, dd (1.5, 8.8)	Ser-NH, Pro^{I}-2, Pro^{I}-3b	Pro^{II}-1, Pro^{II}-5
	3	31.8, CH2	a	2.16, m		Pro^{II}-1
			b	2.00, m	Ser-NH	
	4	21.7, CH2	a	1.77, m	Pro^{III}-3a	
			b	1.57, m		
	5	46.8, CH2	a	3.60, ddd (1.5, 8.4, 10.8)	Ser-NH	
			b	3.33, ddd (10.8, 10.8, 7.1)	Pro^{III}-4b	Pro^{I}-1
Ser	NH			7.65, d (5.9)	Tyr-NH, Pro^{I}-2, Pro^{II}-2, Pro^{II}-5a, Pro^{II}-4b	Pro^{II} -1
	1	167.7, C		-		
	2	60.0, CH		3.85, ddd (3.6, 5.9, 10.2)	Tyr-NH	Ser -1
	3	60.9, CH_2	a	3.46, dd (10.2, 11.9)	Tyr-NH, Tyr-5/9	
			b	3.14, dd (11.9, 3.6)		
Tyr	NH			7.34, d (9.1)	Ser-NH, Ser-3a	Ser-1
	1	171.5 C		-		
	2	51.5 CH		4.88 ddd (3.2, 9.1,10.9)	Pro^{III}-5a/b	Tyr- 1
	3	37.0 CH2	a	3.35, dd (3.2,13.5)	Pro^{III}-5a/b, Phe-NH, Gln-NH	Tyr-5/9
			b	2.42, dd (10.9, 13.5)	Gln-NH, Phe-NH, Pro^{III}-5a	Tyr-1, Tyr-4
	4	126.6 C		-		
	5/9	130.5 CH		7.08, d (8.5)	Tyr-3a, Tyr-3b	Tyr-7
	6/8	114.9 CH		6.66, d (8.5)		Tyr-7
	7	156.0 C		-		
	7-OH			7.42, s		
Pro^{III}	1	171.9, C		-		
	2	63.1, CH		4.06, t (8.7)	Phe-NH,	Pro^{III}-1
	3	28.7, CH_2	a	2.22 m		
			b	1.81, m	Gln-NH	Pro^{III}- 1
	4	25.0, CH_2	a	2.11, m	Gln-NH, Gln-3a, Gln-3b	
			b	1.98, m		
	5	46.9, CH_2	a	3.93, ddd (6.8, 9.8, 9.8)	Tyr-2, Tyr-3a	
			b	3.82, m	Tyr-2, Gln-NH, Tyr-3a	Pro^{III} -3
Gln	NH			8.17, d (7,0)	Phe-NH, Tyr-3a, Pro^{III}-2, Pro^{III}-3b, Pro^{III}-4a	Pro^{III}-1
	1	170.7, C		-		
	2	52.8, CH		4.05, ddd (4.3, 7.0, 10.0)	Phe-NH, Pro^{III}-4b	Gln-1
	3	25.9, CH_2	a	1.85, m	Pro^{III}-4a	Gln-1, Gln-5
			b	1.73, m	Pro^{III}- 4a	Gln-5
	4	31.5, CH_2	a	2.13, ddd (7.2, 15.7, 7.2)	Pro^{III}-5b, Pro^{III}-3a, Pro^{III}-3b	Gln-5
			b	2.04, ddd (7.2, 15.7, 7.2)	Pro^{III}-4a	Gln-5
	5	174.5, C		-		
	5-NH_2			6.92, s	Gln-4a, Gln-4b	Gln-5
Phe	NH			7.11, d (7.2)	Phe-2, Gln-NH, Pro^{III}-2, Pro^{I}-5b, Tyr-NH	Gln-1
	1	167.5 C				
	2	51.5, CH		4.69, ddd (5.8,7.2, 8.0)	Pro^{I}-5a, Pro^{I}-5b	Phe-1, Phe-4
	3	36.9 CH_2	a	3.18, dd (8.0, 14.2)		Phe-1, Phe-4
			b	2.71 (5.8, 14.2)		Phe-1, Phe-5/9
	4	138.0, C		-		
	5/9	128.9, CH		7.16, d (7.5)		
	6/8	126.0, CH		7.18, t (7.3)		
	7	128.0, CH		7.22, t (7.5)		

Table S3. Links to LC-MS data and molecular networks.

Description	Link
Classical Molecular Networking (Metabolomics) workflow	https://gnps.ucsd.edu/ProteoSAFe/status.jsp?task=4acb49028d7041c39fccf03dd6e8c195
Feature-Based Molecular Networking workflow	https://gnps.ucsd.edu/ProteoSAFe/status.jsp?task=db7aa9e8bec64d6290cc040254f00b87
Dereplicator	https://gnps.ucsd.edu/ProteoSAFe/status.jsp?task=f8b123b5ea89494481549c1a519caf5d
LC-MS data on Massive	https://massive.ucsd.edu/ProteoSAFe/dataset.jsp?task=f77c49cb5d73489583c08d3e66b558e1<br>FTP access: ftp://MSV000085904@massive.ucsd.edu<br>password: Sty_reviewers

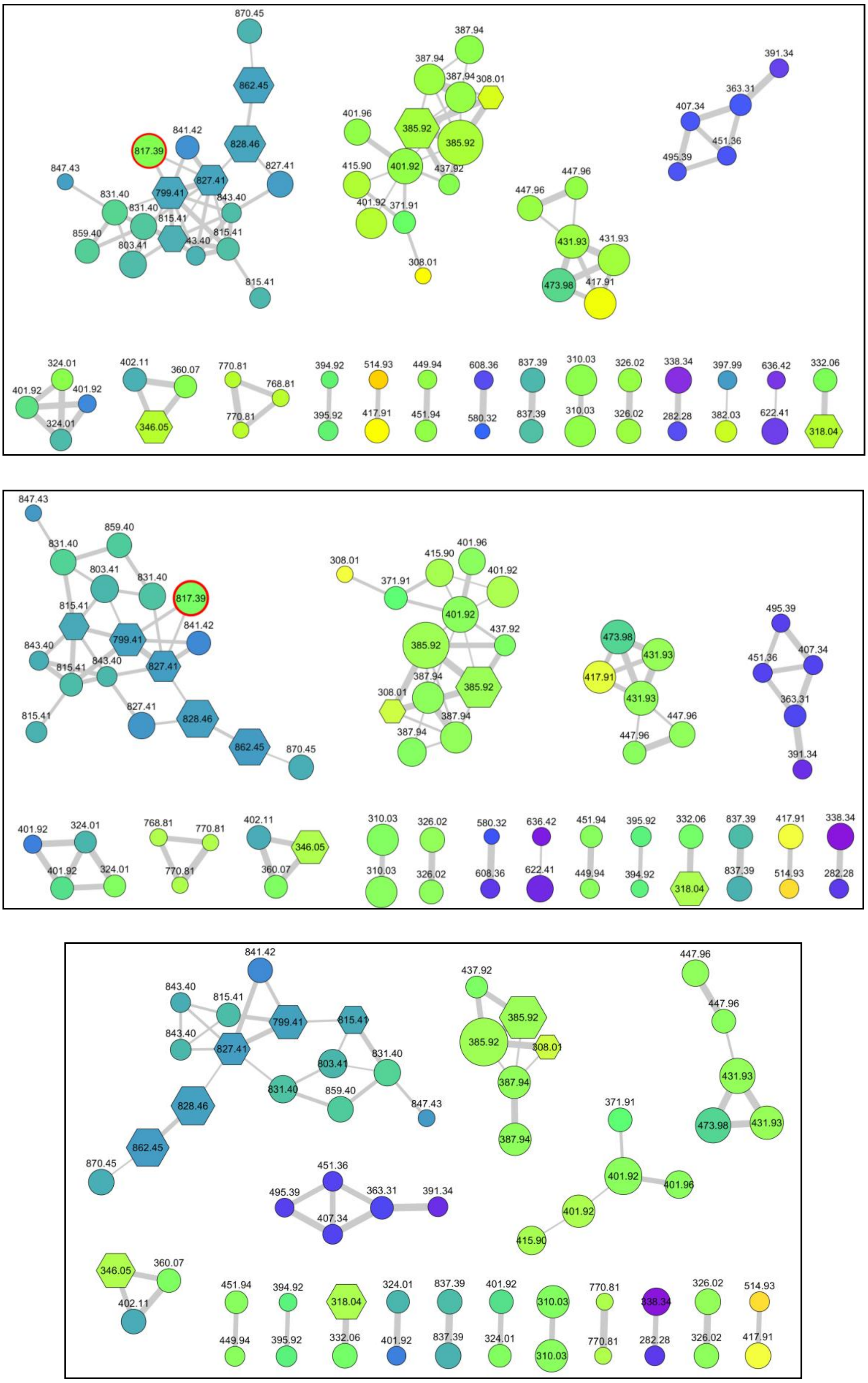

Figure S1. Molecular networks of the extract of *S. caribica* obtained with the same data and the same parameters (*m*/*z* tolerance 0.01 Da, cosine score > 0.55, matched peaks > 8, maximum number of neighbor nodes = 10) using (a) the program MetGem (b) the Metabolomics workflow on GNPS, and (c) the Feature-Based Molecular Network workflow on GNPS.

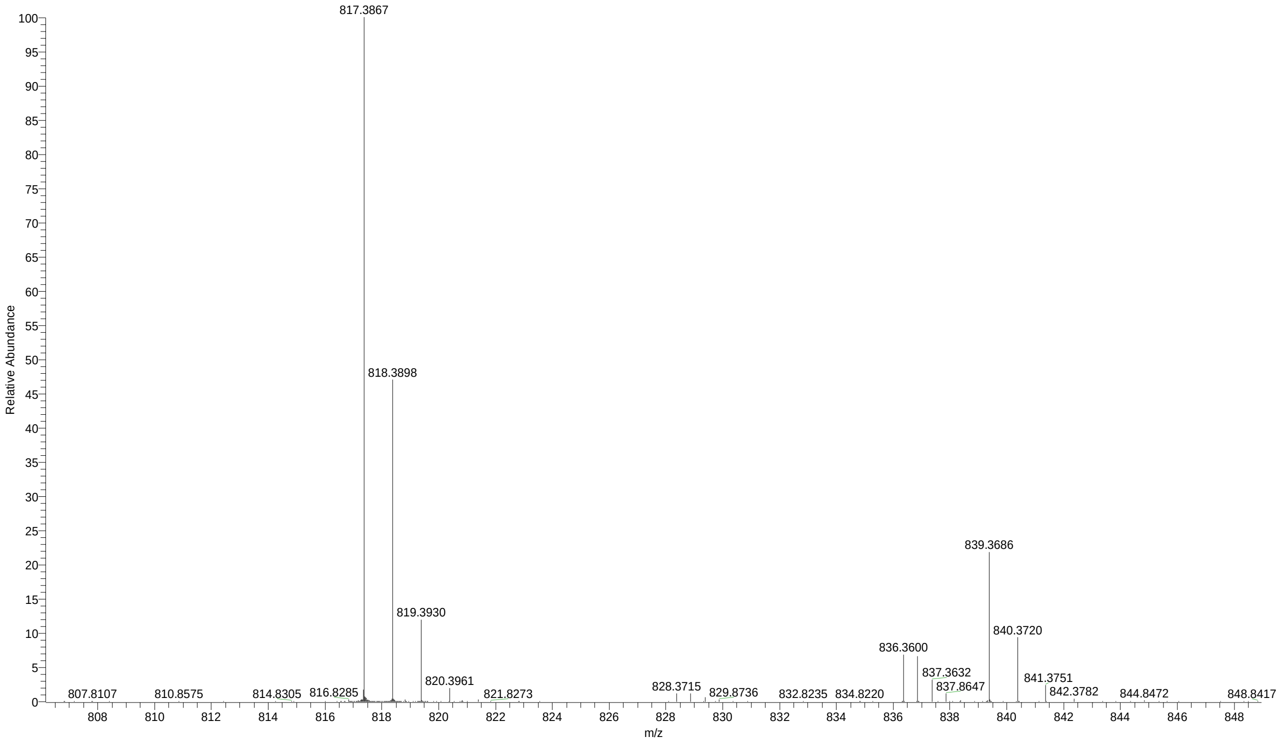

Figure S2. Positive ion mode high-resolution ESI mass spectrum of stylissamide L (1).

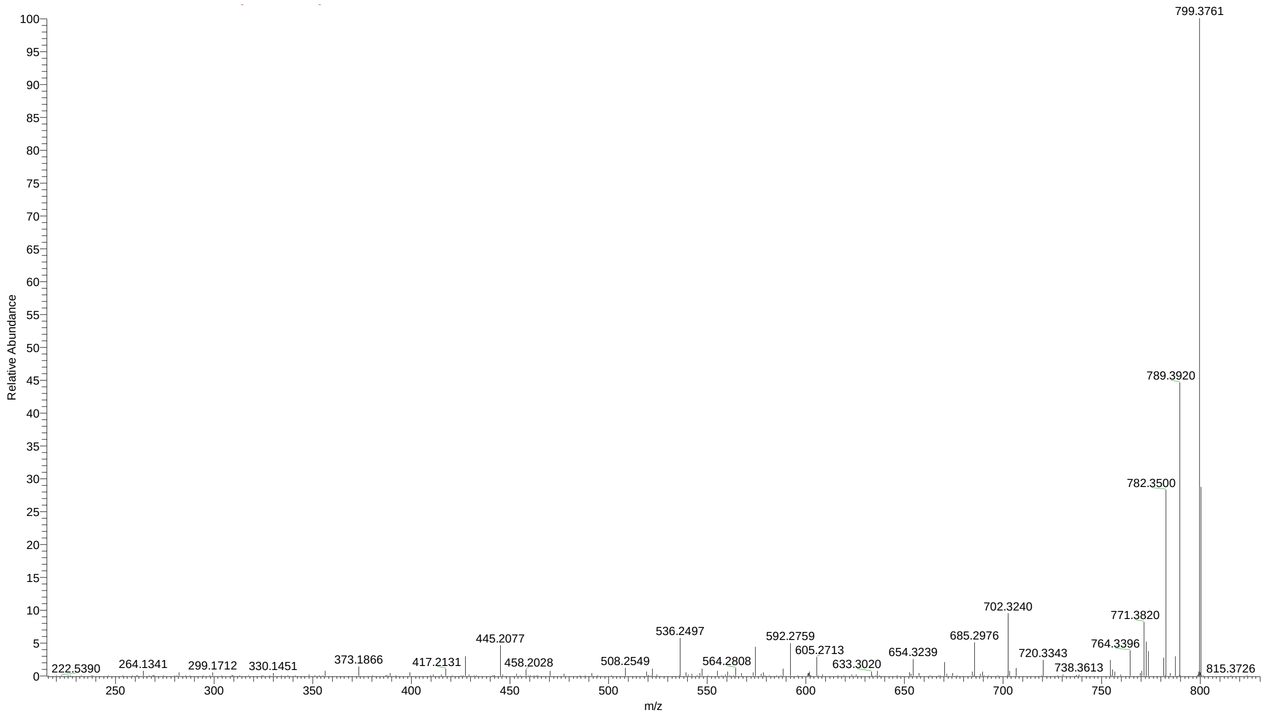

Figure S3. Positive ion mode high-resolution ESI MS/MS spectrum of stylissamide L (1).

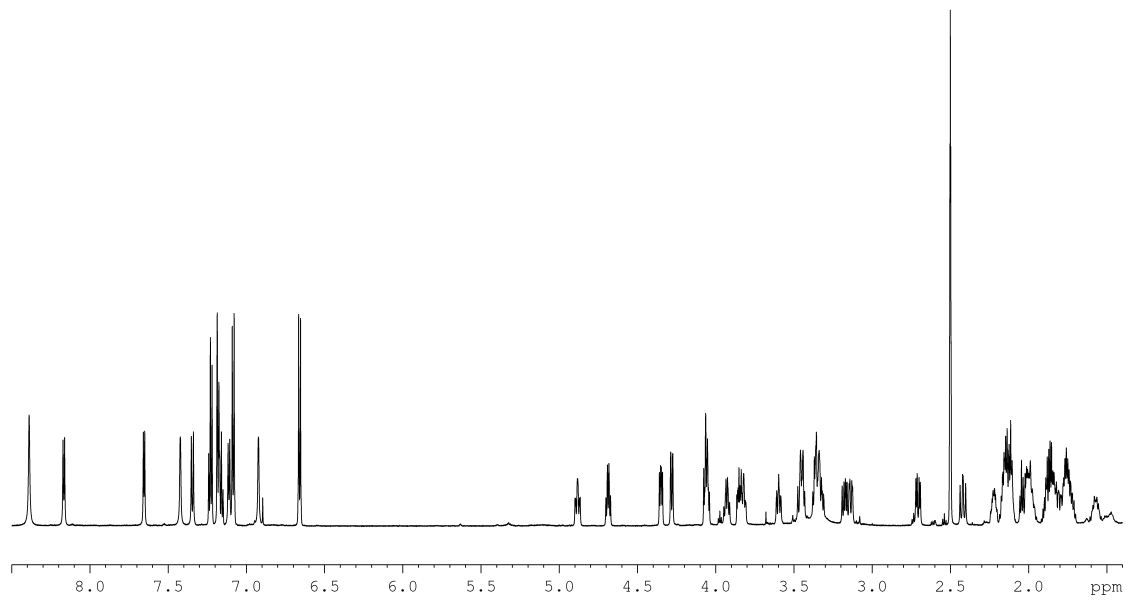

Figure S4. ^{1}H-NMR spectrum of stylissamide L (1) (700 MHz, DMSO-d_6).

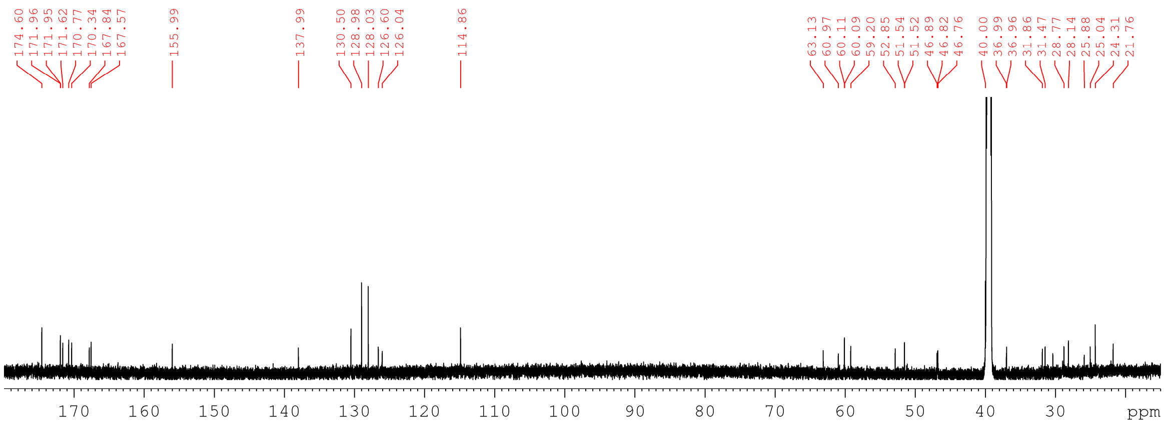

Figure S5. ^{13}C-NMR spectrum of stylissamide L (1) (175 MHz, DMSO-d_6).

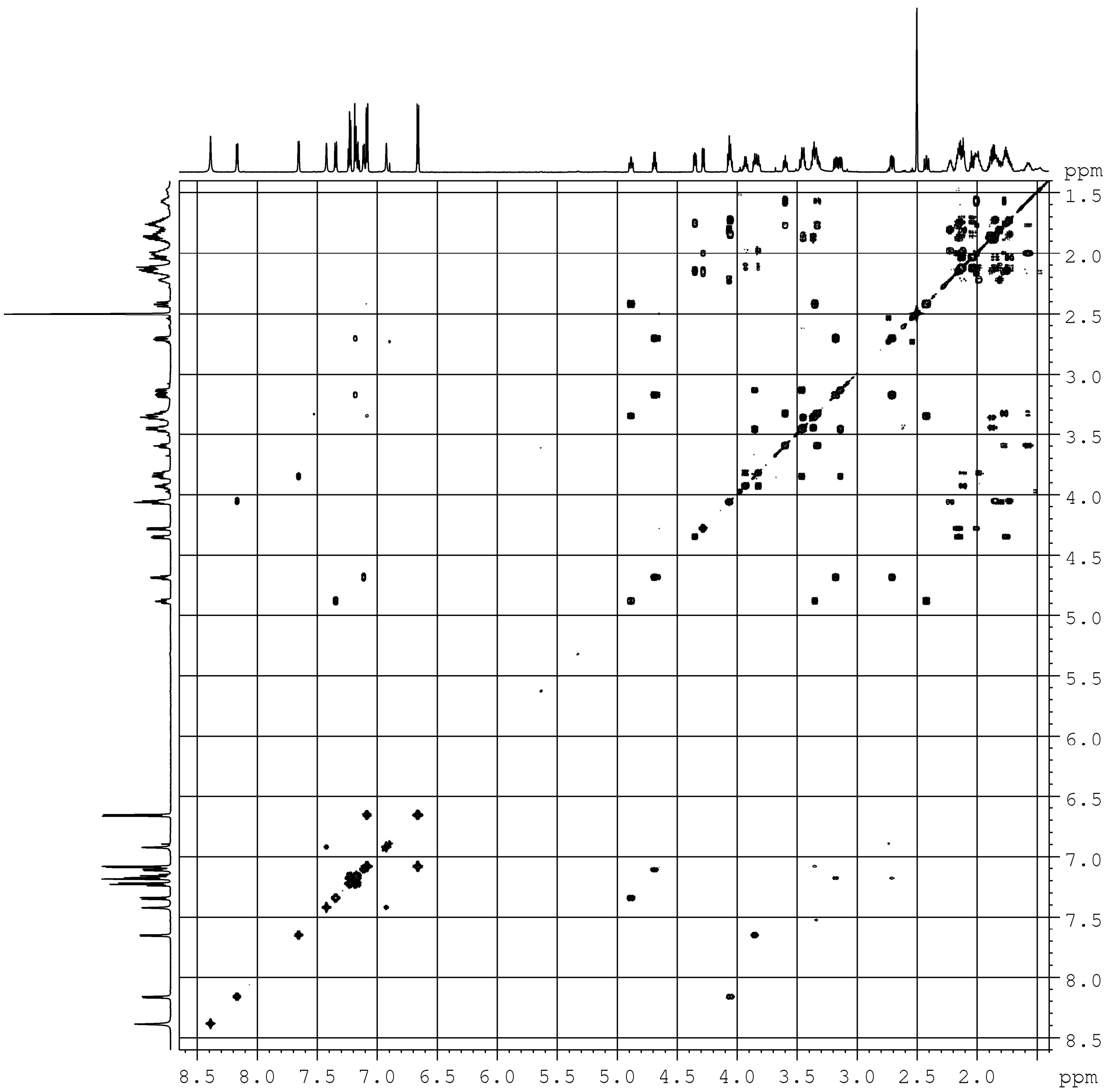

Figure S6. COSY spectrum of stylissamide L (1) (700 MHz, DMSO-d_6).

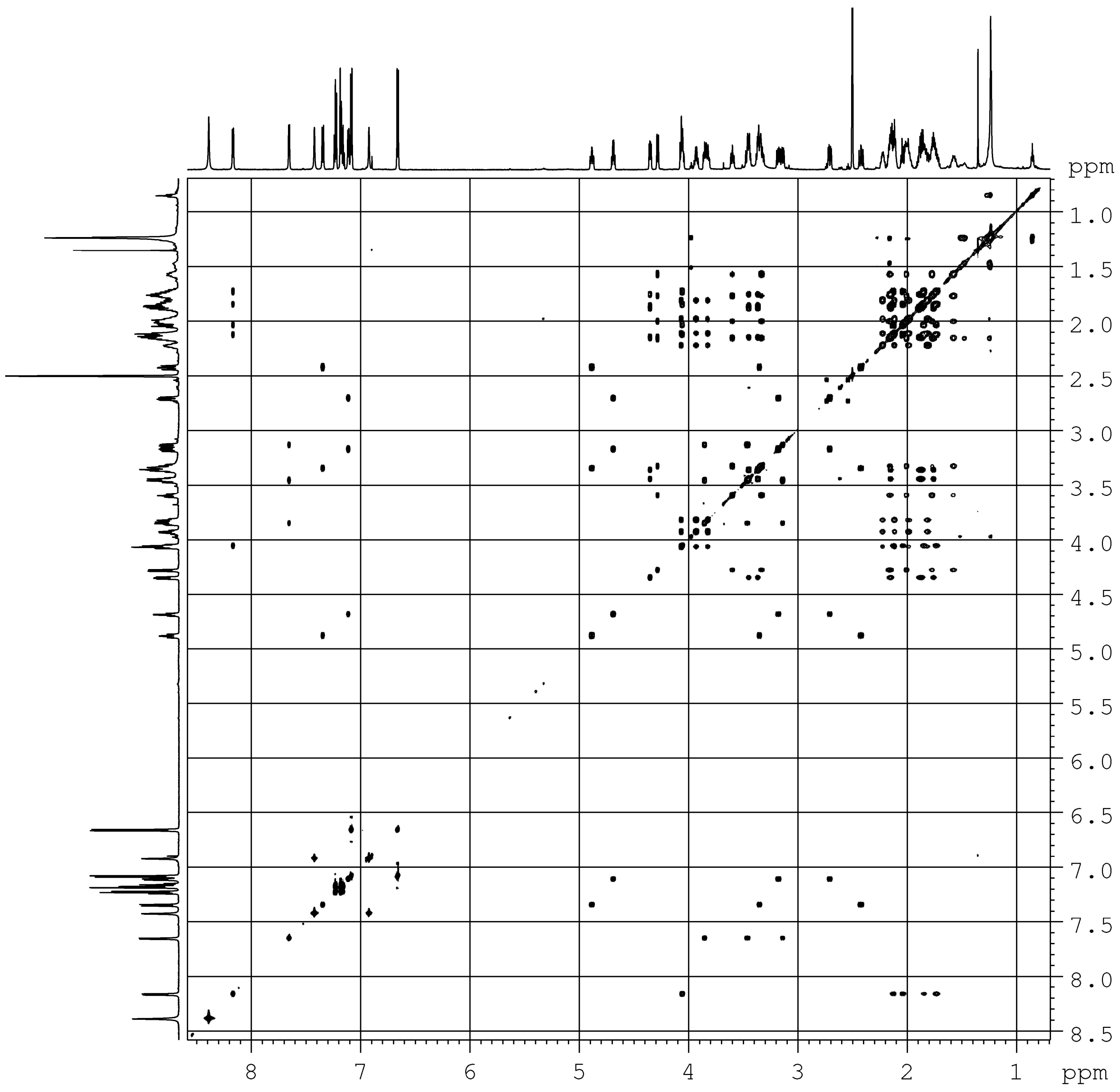

Figure S7. TOCSY spectrum of stylissamide L (1) (700 MHz, DMSO-d_6).

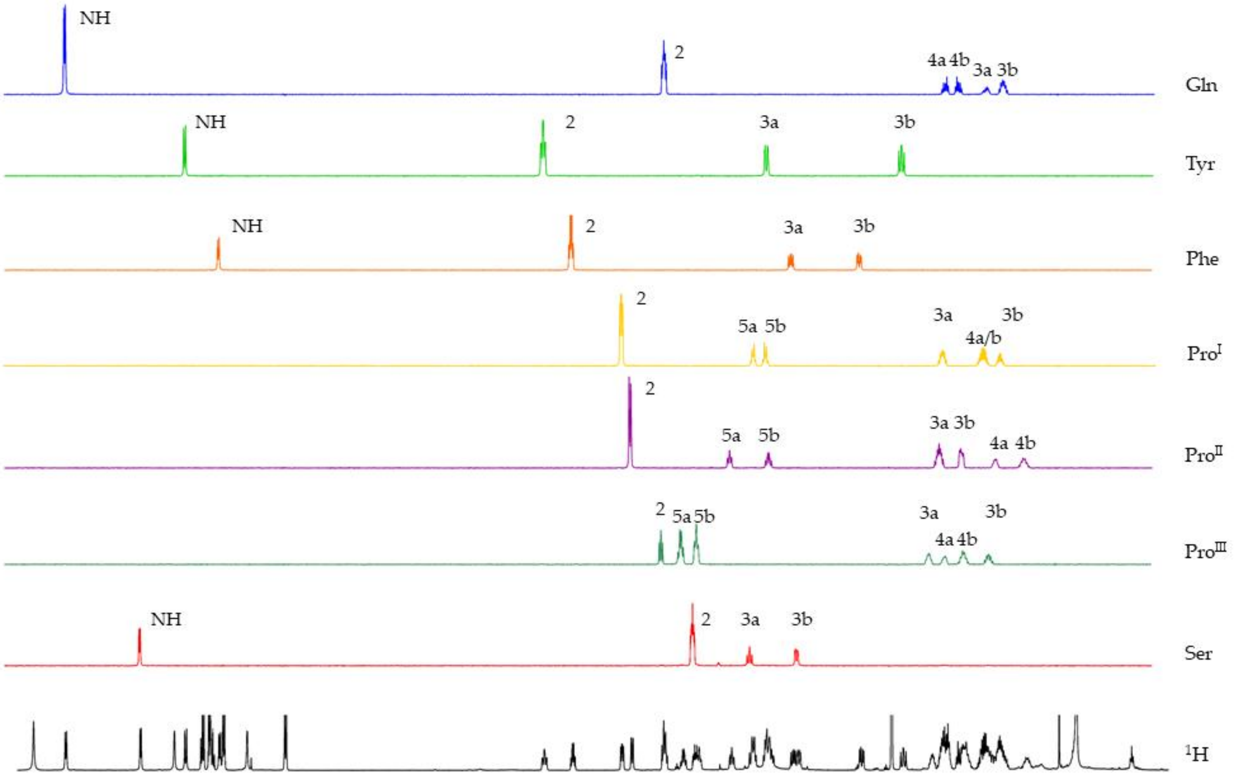

Figure S8. Spin systems of stylissamide L (1) from the sections of the TOCSY spectrum.

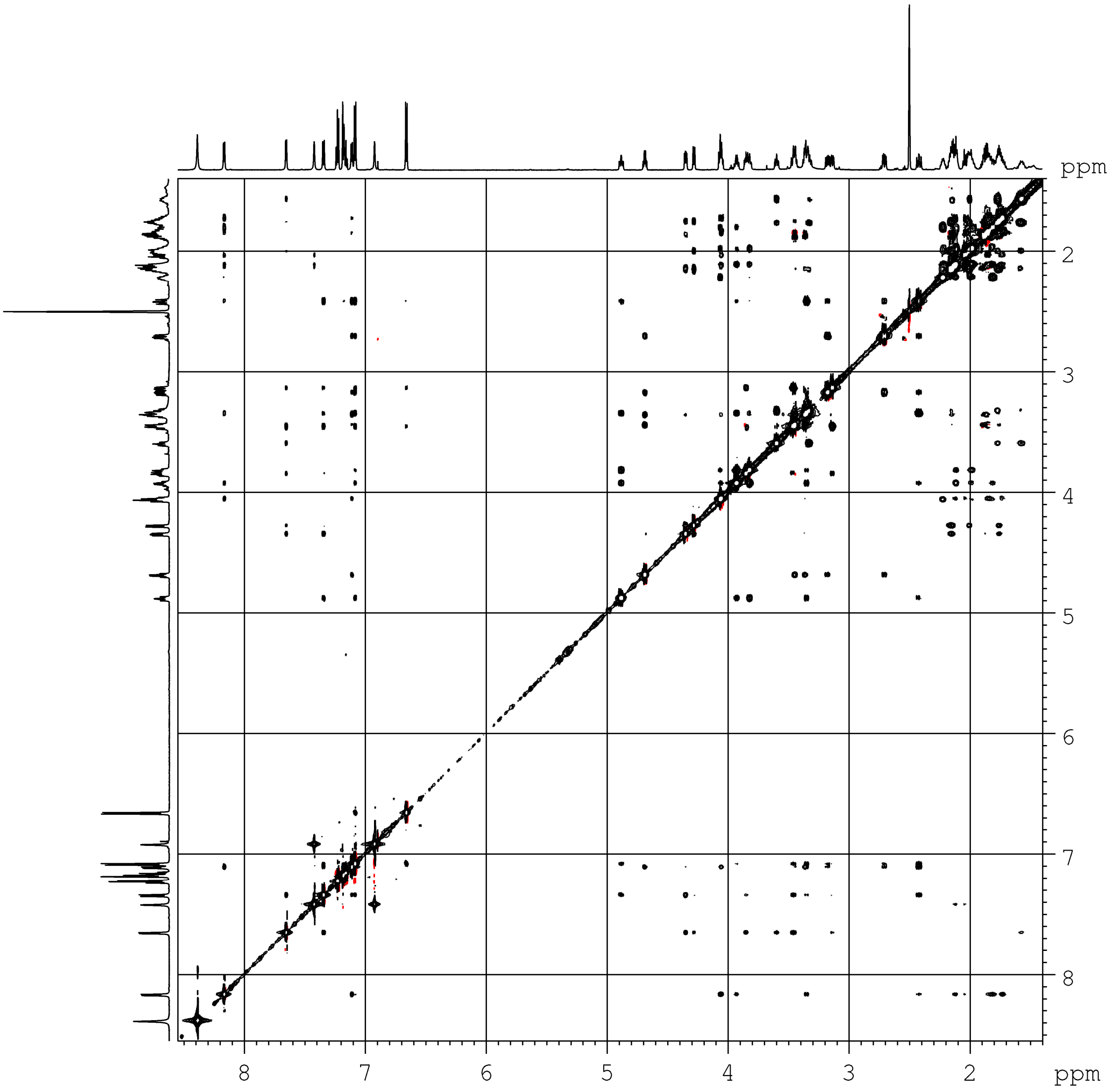

Figure S9. NOESY spectrum of stylissamide L (1) (700 MHz, DMSO-d_6).

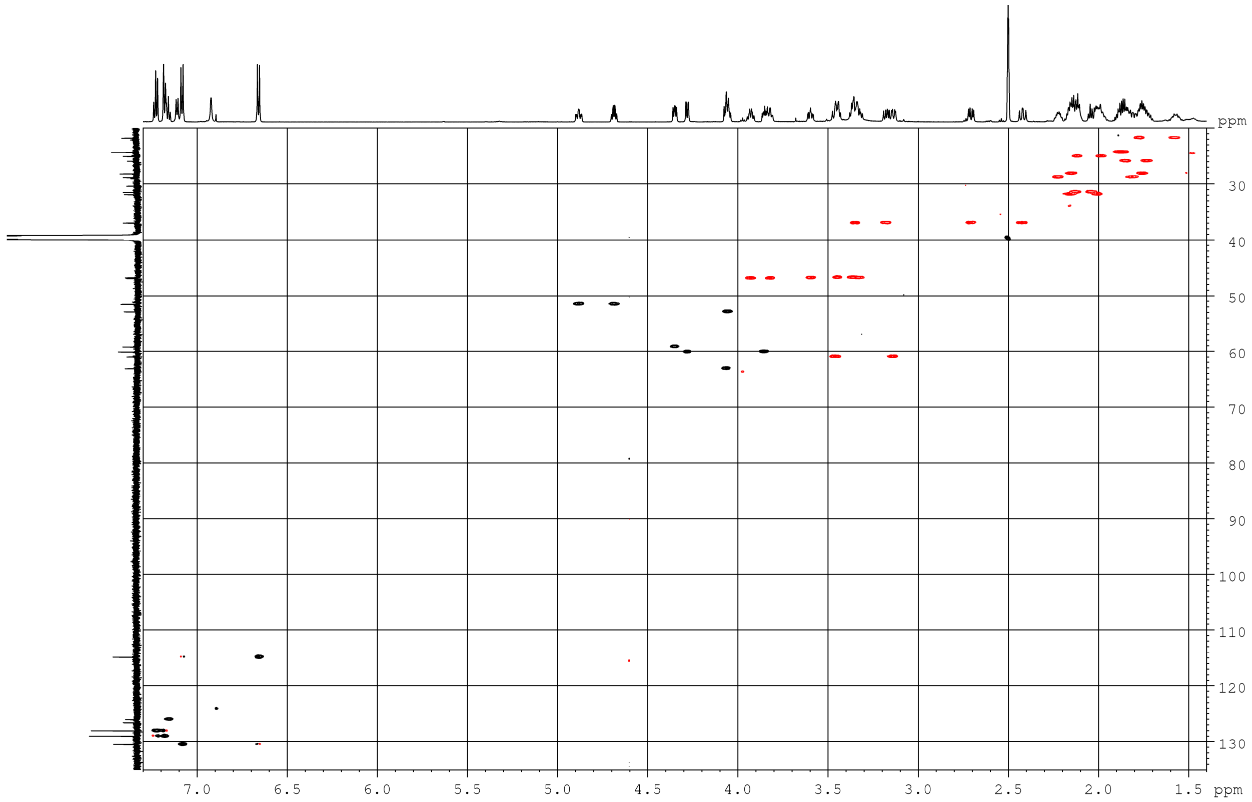

Figure S10. HSQC spectrum of stylissamide L (1) (700 MHz, DMSO-d_6).

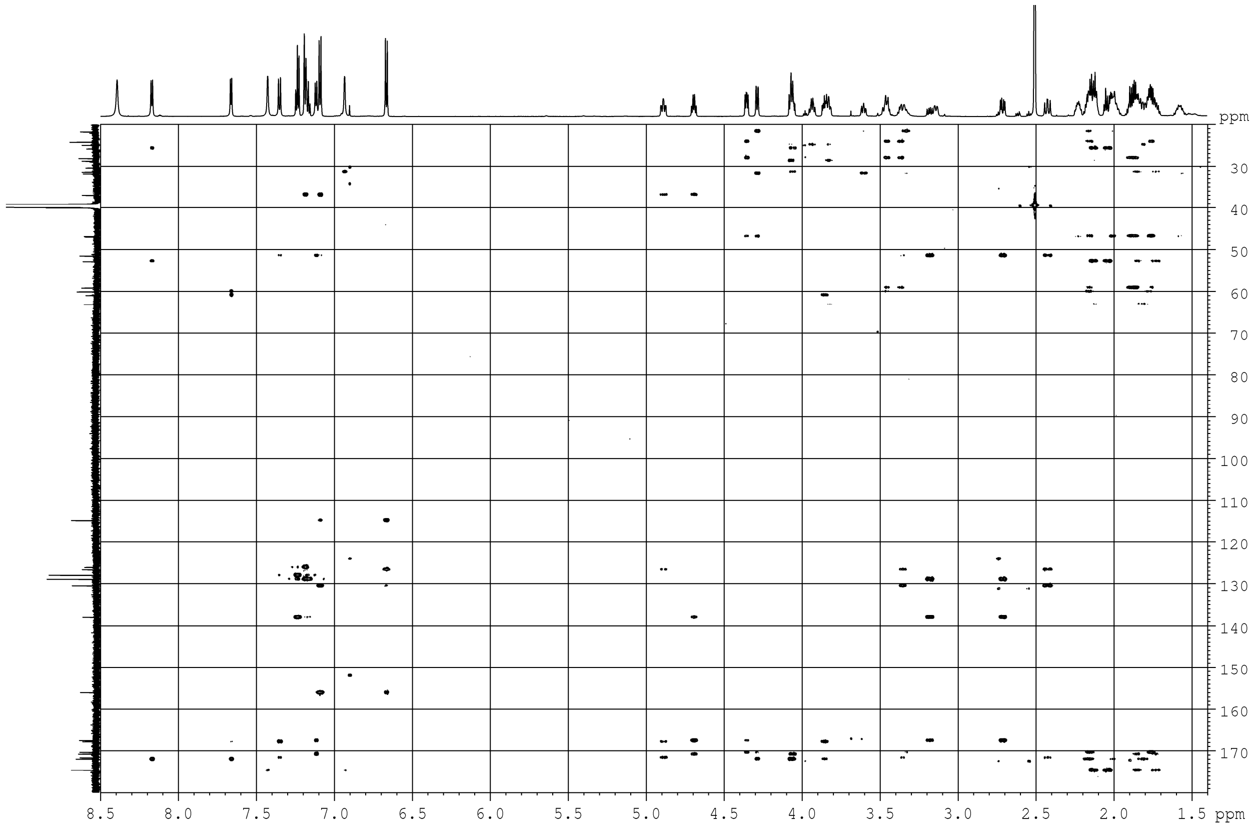

Figure S11. HMBC spectrum of stylissamide L (1) (700 MHz, DMSO-*d*$_6$).

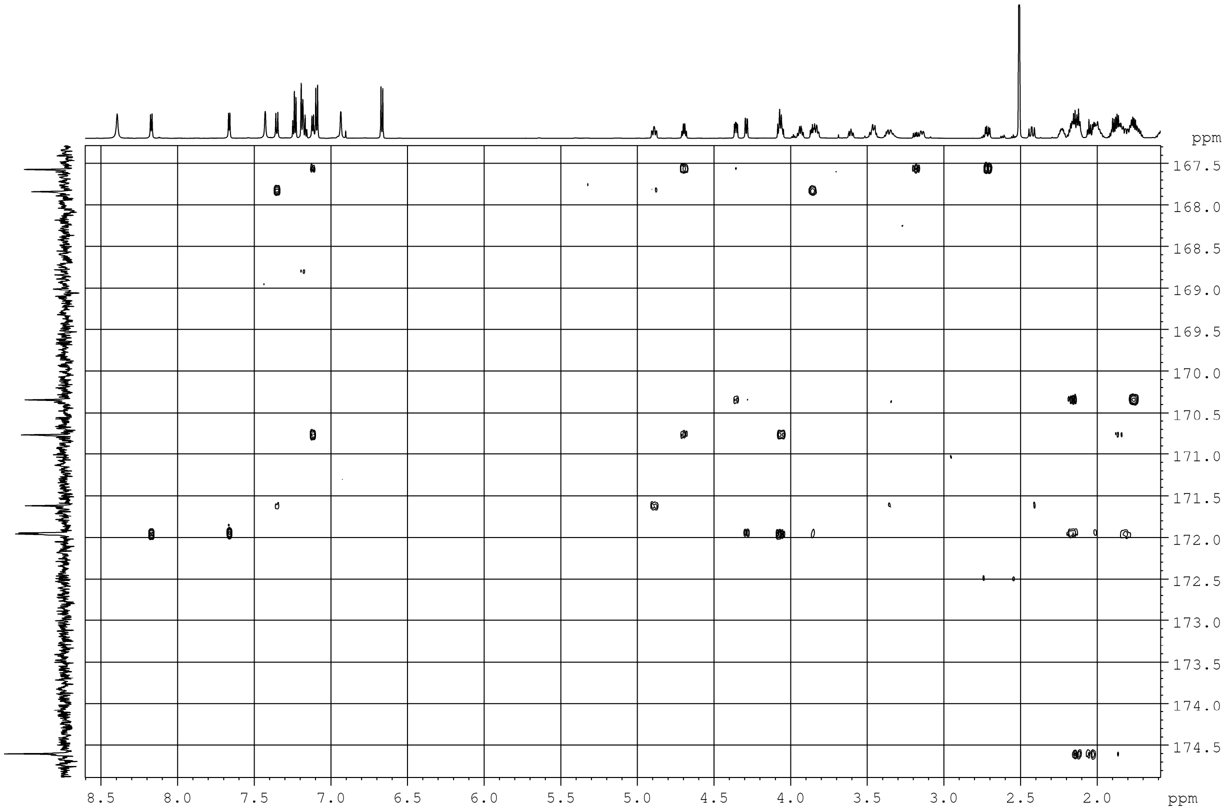

Figure S12. Band-selective HMBC spectrum of stylissamide L (1) (700 MHz, DMSO-d_6).

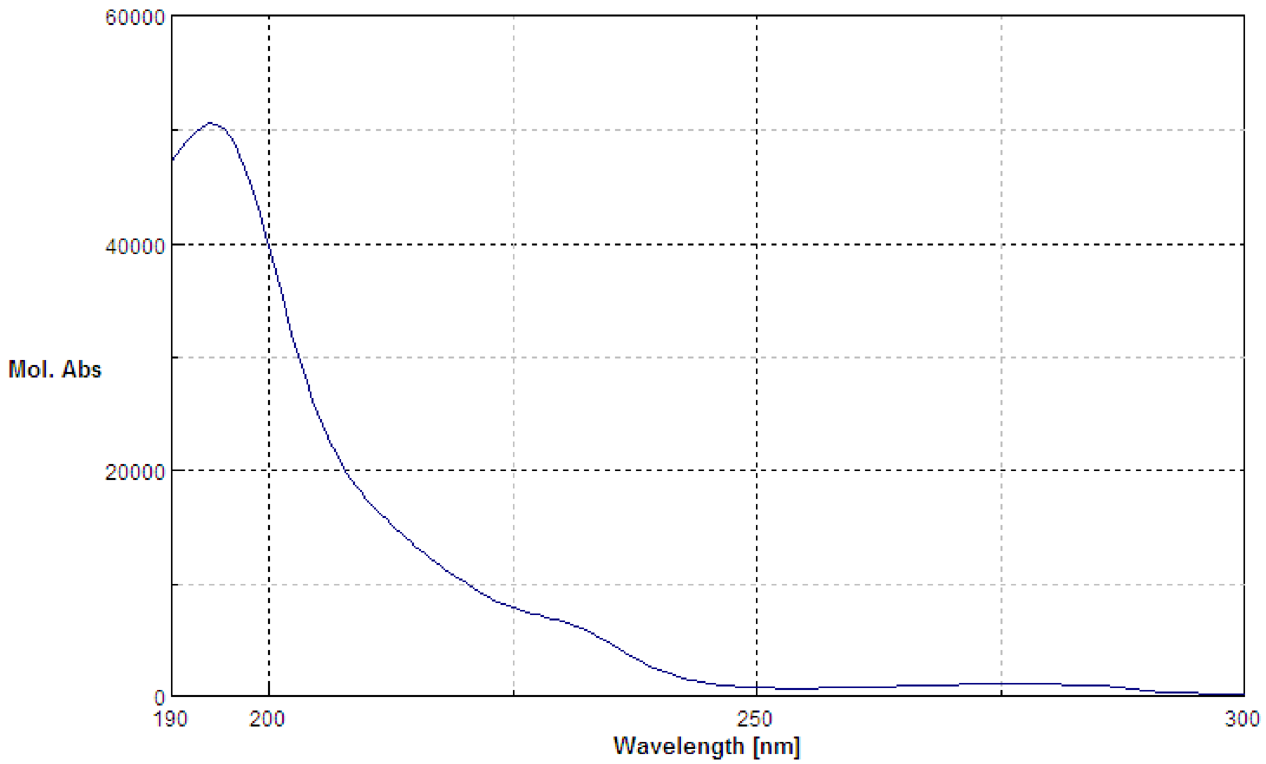

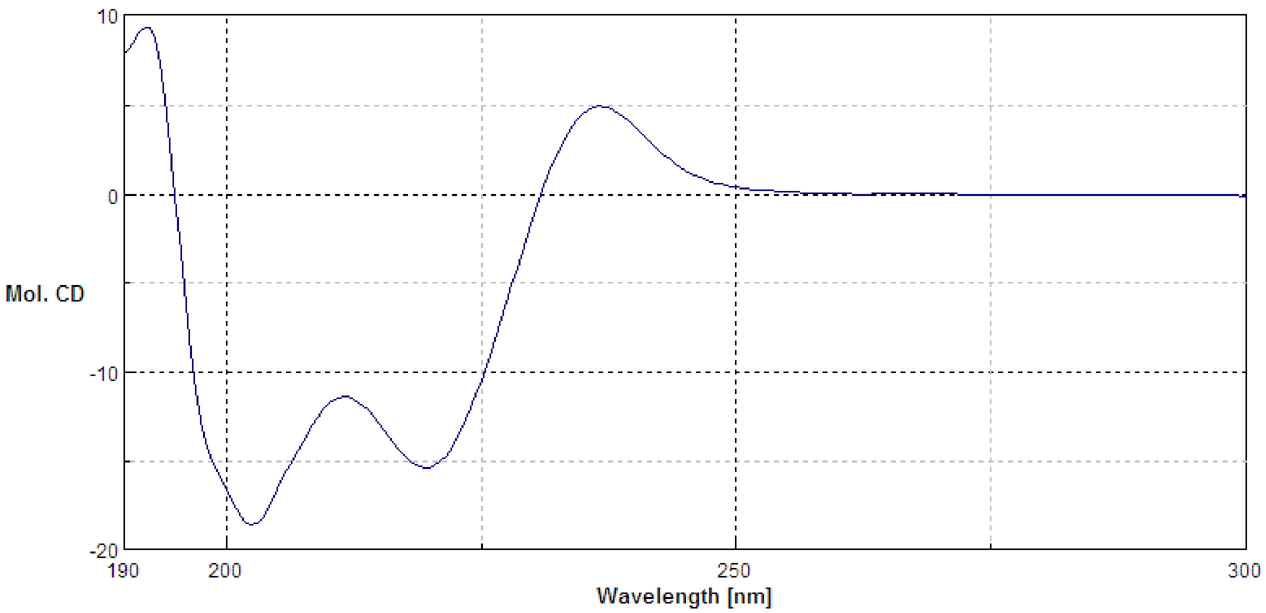

Figure S13. UV and ECD spectra of stylissamide L (1) in ACN.

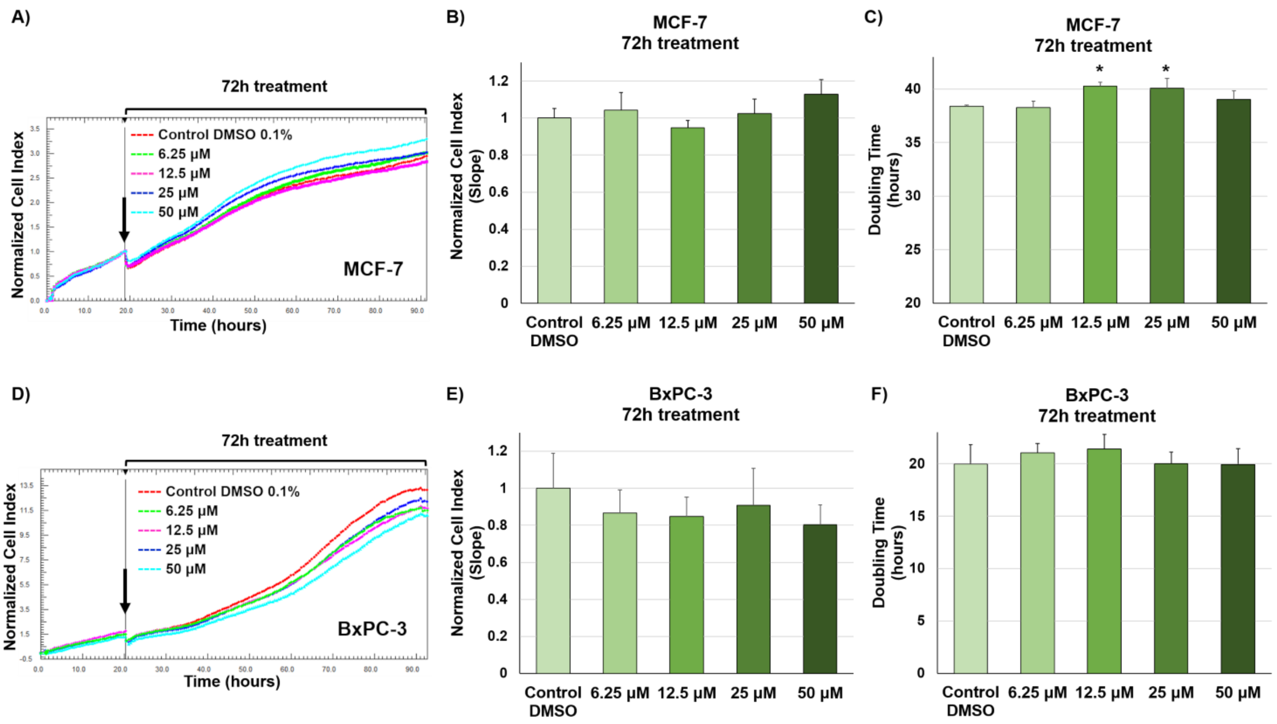

Figure S14. Real time monitoring of cancer cell proliferation after exposure to stylissamide L (**1**) and DMSO vehicle, by using the xCELLigence System Real-Time Cell Analyzer. (**A**,**D**) Normalized cell index (NCI) traces of MCF7 (**A**) and BxPC-3 (**D**) cells treated with different concentrations (6.25, 12.5, 25, and 50 μM) of stylissamide L (**1**) and DMSO vehicle (0.1%) for 72 hours. Black arrow shows the start of drug treatment. Each cell index value was normalized at this time. (**B**,**E**) Slope values of growth curves of MCF-7 (**B**) and BxPC-3 (**E**) cells after 72 h exposure to different concentrations (6.25, 12.5, 25, and 50 μM) of stylissamide L (**1**) and DMSO vehicle (0.1%). NCI slope values are relative to controls treated with DMSO vehicle. (**C**,**F**) Doubling times of NCI of MCF-7 (**C**) and BxPC-3 (**F**) cells after 72 h treatment with different concentrations (6.25, 12.5, 25, and 50 μM) of stylissamide L (**1**) and DMSO (0.1%). Data are presented as mean ± SD; n=3. Statistical significances are referred to the DMSO control. One-way analysis of variance (ANOVA) was applied to compare means of groups and Dunnett's method was used as a post-hoc test to compare multiple groups versus the control group. p-values < 0.05 were considered to be statistically significant. Statistical analysis was performed using the GraphPad Prism Software Version 5. * $p < 0.05$.

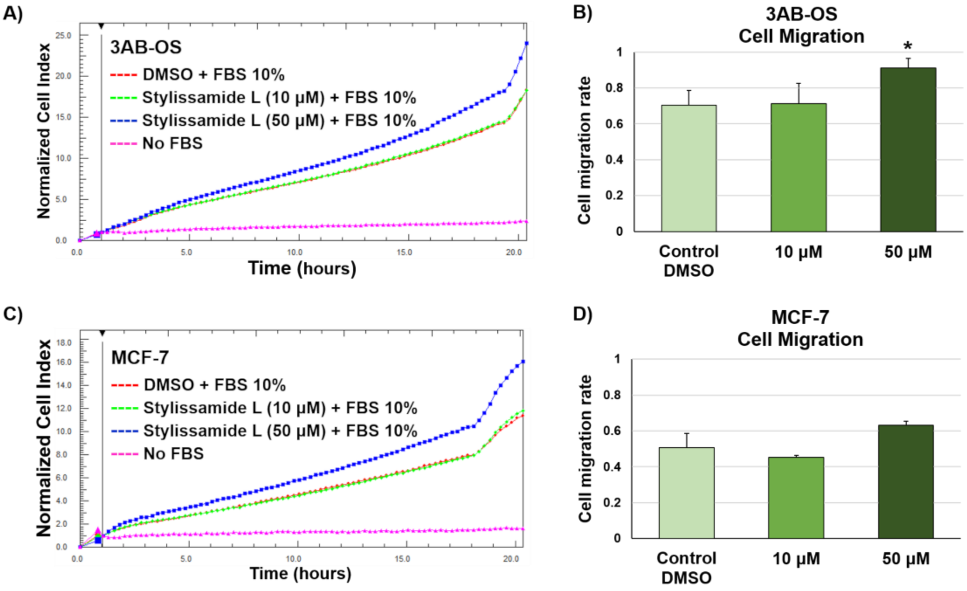

Figure S15. Real-time monitoring of 3AB-OS and MCF-7 cell migration after exposure to stylissamide L (**1**). (**A**,**C**) NCI traces of 3AB-OS (**A**) and MCF-7 (**C**) cells seeded with compound **1** or DMSO (0.1%) vehicle, in presence of 10% Fetal Bovine Serum (FBS) as the chemoattractant. Migration was monitored for 20 hours, using the xCELLigence System equipped with specially designed 16-well plates (CIM-plate 16). (**B**,**D**) Migration activity of 3AB-OS (**B**) and MCF-7 (**D**) cells seeded with compound **1** or DMSO (0.1%) vehicle, in presence of 10% Fetal Bovine Serum (FBS) as the chemoattractant. Cell migration rates were recorded for 20 hours and expressed as slope values of NCI curves. Data are presented as mean ± SD; n=3. Statistical significances are referred to the DMSO control. Two-group comparisons were performed using Student's t-test. P-values < 0.05 were considered to be statistically significant. Statistical analysis was performed using the GraphPad Prism Software Version 5.
